# Remote training of behavioral intervention facilitators: model development and lessons learned

**DOI:** 10.3389/fdgth.2026.1768119

**Published:** 2026-07-31

**Authors:** Yong Gun Lee, Vitaliy Vinogradov, Ethan H. Freedman, Kelsey G. Reeder, Alissa Davis, Emily Allen Paine, Caitlin I. Laughney, Sultana Valentina Káli, Aidar Yelkeyev, Karina Alipova, Gaukhar Mergenova, Xiaotong Yang, Sholpan Primbetova, Assel Terlikbayeva, Elwin Wu

**Affiliations:** 1 Department of Social Work and Social Administration, The University of Hong Kong, Pokfulam, Hong Kong SAR, China; 2 Social Intervention Group, School of Social Work, Columbia University, New York, NY, United States; 3 Global Health Research Center of Central Asia, Columbia University, Almaty, Kazakhstan; 4 Departments of Psychiatry and Sociomedical Sciences, Columbia University, New York, NY, United States; 5 Division of Community Engagement and Implementation Science, New York State Psychiatric Institute, New York, NY, United States; 6 Epidemiology Department, Asfendiyarov Kazakh National Medical University, Almaty, Kazakhstan; 7 Department of Sociology and Social Work, Farabi University, Almaty, Kazakhstan

**Keywords:** behavioral intervention, clinical trials, HIV/AIDS, Kazakhstan, remote training, sexual and gender diversity

## Abstract

**Introduction:**

Intervention facilitator training is critically important for successful implementation of complex behavioral interventions. As remote intervention delivery becomes more common, remote training, whereby training components are deployed using digital technology, has emerged as an alternative to traditional in-person training. However, evidence-based guidelines remain limited for implementing remote training tailored to remote intervention delivery.

**Materials & methods:**

We developed and applied the Groundwork-Implementation-Feasibility (GIF) model to the remote training of facilitators preparing to remotely deliver *PRIDE in HIV Care*, an HIV prevention intervention for sexually diverse and gender expansive populations in Kazakhstan.

**Results:**

Guided by the GIF model, the remote facilitator training included formative assessment and planning, a five-day workshop featuring demonstration and real-play exercises, and ongoing individualized feedback. All facilitators completed the workshop with high satisfaction. Remote training increased access to digital training materials and enabled the development of remote-specific skills. By engaging in the training remotely themselves, facilitators gained parallel process insight that helped them anticipate challenges faced by intervention recipients, including technical difficulties, privacy concerns, and unstable connection.

**Discussion:**

Overall, remote intervention facilitator training for remote intervention delivery offers advantages beyond cost reduction, requires remote-specific skills, and leverages parallel processes to strengthening technical, content, and engagement abilities. While the GIF model provides a framework for developing future remote training programs, the growing use of technology in behavioral health interventions underscores the need for training that prepares facilitators for the technical, interpersonal, and ethical complexities of digital intervention delivery.

**Clinical Trial Registration:**

https://clinicaltrials.gov/study/NCT02786615, identifier NCT02786615.

## 1 Introduction

Behavioral health interventions are primary strategies for intervening in health and psychosocial issues ranging from chronic disease management to infectious disease prevention. Over the past two decades, research has rigorously demonstrated that behavioral interventions have clinically meaningful effectiveness—thus termed evidence-based interventions (EBIs)—in improving health outcomes (1, 2). Systematic, structured, and sufficient training of individuals tasked with delivering EBIs (i.e., intervention facilitators) constitutes essential steps in the research, delivery, and sustainability of EBIs (3–6). Intervention training is also invaluable if/when EBIs need to be adapted for different geographical locations or cultural contexts, social trends (e.g., increasing use of digital technology has led to increased accessibility and popularity of eHealth and mHealth modalities), and external, catastrophic events that change the intervention delivery environment, such as natural disasters, wars, and pandemics (7–9).

The COVID-19 pandemic, more specifically its mitigation measures like social distancing requirements, travel restrictions, and facility closures, disrupted research and service delivery across health and social service sectors globally. Efforts to maintain rigor, integrity, and consistent care catalyzed the adaptation of interventions requiring physical presence to remote formats supported by digital platforms—for instance, expanding engagement through telecommunication and virtualization (10–15). This transition was especially critical for behavioral health interventions serving socially marginalized and vulnerable populations, as service disruptions could exacerbate barriers like stigma around identity disclosure, heightened concerns about confidentiality and anonymity, and new fear of exposure to SARS CoV2 (16–19). This transition also called for heightened attention to the safety of all intervention recipients (20). Consequently, for the rapid transformation of intervention delivery modalities necessitated facilitator trainings to enhance competence and motivation in delivering interventions remotely (21, 22).

Remote training of intervention facilitators, in which training components are deployed using digital technology and trainers and trainees are in spatially disparate locations, is not new. Extant research has demonstrated that, across health and social care domains, remote intervention facilitator was not only accessible, feasible, and effective, but also beneficial in many regards including accessibility, cost-effectiveness, and scalability (23–27). In addition, when training involves synchronous interaction, the remote modality may offer a distinct advantage over the traditional, in-person modality of simulating the environment of remote intervention delivery and enabling intervention facilitators to experience and anticipate parallel scenarios, opportunities, and challenges (28). While models guiding systematic implementation of remote intervention facilitator training have been developed (29, 30), they do not adequately address the competencies in remote intervention delivery. Guidelines for developing and implementing remote training for facilitators who deliver interventions remotely remain limited. Furthermore, there is a paucity of data on remote intervention facilitator training for remote intervention delivery, especially in lower- to middle-income countries.

The objective of this paper is to describe in detail a process of developing and implementing remote intervention facilitator training for remote delivery of *Peer Reach and Influencer-Driven Engagement in HIV Care Continuum* (*PRIDE in HIV Care* for short), a crowdsourcing and peer-actuated network intervention for increasing engagement of sexually diverse and gender expansive (SDGE) individuals in the HIV care continuum in Kazakhstan. By outlining the steps and strategies involved and presenting lessons learned, this paper aims to inform future intervention science efforts conducting remote intervention facilitator training for remote intervention delivery.

## 2 Materials and methods

### 2.1 Parent study design

The clinical trial of *PRIDE in HIV Care* (ClinicalTrials.gov Identifier: NCT02786615) used a stepped wedge design in which the intervention was rolled out sequentially across three major cities of Kazakhstan: Almaty, Shymkent, and Astana (31). These cities were estimated to have the highest numbers of SDGE individuals and to experience rapid growth in new HIV infections nationally (32, 33). After a period of data collection without any intervention (July 2018 – February 2019), the intervention was sequentially implemented in each study city, spaced six months apart: March 2019 in Almaty; August 2019 in Shymkent; and March 2020 in Astana. During a city's implementation phase, the intervention was offered to interested and consenting study participants in that city. Informed consent was obtained from all study participants at the time of enrollment in the trial. The study procedures were approved by the Institutional Review Boards of Al-Farabi Kazakh National University and Columbia University.

### 2.2 Intervention

*PRIDE in HIV Care* was designed and manualized by the study's Principal Investigator and Project Directors (the ‘developers’) and delivered to study participants by trained intervention facilitators (the ‘facilitators’). Facilitators were research study team members who had completed compulsory education (i.e., secondary education) and possessed experience with behavioral health research and/or SDGE community organizing as health consultants or outreach workers; they were not health professionals. Framed within the Network-Individual-Resource model for HIV prevention (34), the intervention aimed to recruit and motivate SDGE individuals in Kazakhstan to engage peers in the HIV care continuum. The intervention also incorporated constructs of social cognitive (35) and social marketing (36) theories in activities designed to promote testing and treatment among intervention recipients and their peers. In its original form, the intervention consisted of four group sessions (target group size of 4–6 study participants) covering the following topics: (1) Crowdsourcing to identify HIV-related care services in one's city of residence and ways of safely accessing them; (2) building skills as a peer influencer in ‘multiplying’ HIV prevention strategies and motivation in various settings (e.g., online vs. offline, public/open vs. private/closed); (3) leveraging existing and emerging technologies for social marketing of testing and treatment; and (4) addressing potential threat to sustaining one's role as a peer influencer. Based on prior feedback from a working group regarding a need for a more gradual introduction to a group setting, an orientation session was added to be delivered to each intervention recipient individually before the start of a group cycle to provide them space to discuss and address expectations, concerns, and comfort and safety issues. All sessions were delivered in-person at a field office by a trained facilitator.

By March 2020, all study cities had entered the implementation phase. At the same time, the outset of the COVID-19 pandemic and mitigation measures (e.g., travel and social restrictions) prompted the study to adapt all in-person activities, including intervention delivery, to a remote format. The process of adapting intervention delivery to a remote format is described elsewhere (15). Briefly, principally due to COVID-19-related restrictions and safety concerns, the intervention was updated and modified to be delivered to single recipients remotely and individually on a teleconferencing platform, such as Zoom. We opted against a group format to minimize the impact of technical difficulties and ensure better protection of intervention recipients’ privacy and confidentiality when using telecommunications technology (37). The Facilitator's Manual was updated to reflect all modifications.

### 2.3 Intervention facilitator training

Modifying the intervention delivery format—from in-person, group to remote, individual—generated new intervention components and implementation strategies for the facilitators to learn. Therefore, we planned for training designed to enhance facilitators’ familiarity with new intervention content, procedures, and techniques used in modified intervention delivery. As the COVID-19 pandemic and mitigation measures persisted, we were forced to plan for remote training as an additional, supplemental component to the prior, in-person training. However, we soon realized there might be advantages of remote training particularly for remote intervention delivery. Thus, we designed remote intervention facilitator training strategically to realize those advantages.

**Figure 1:**
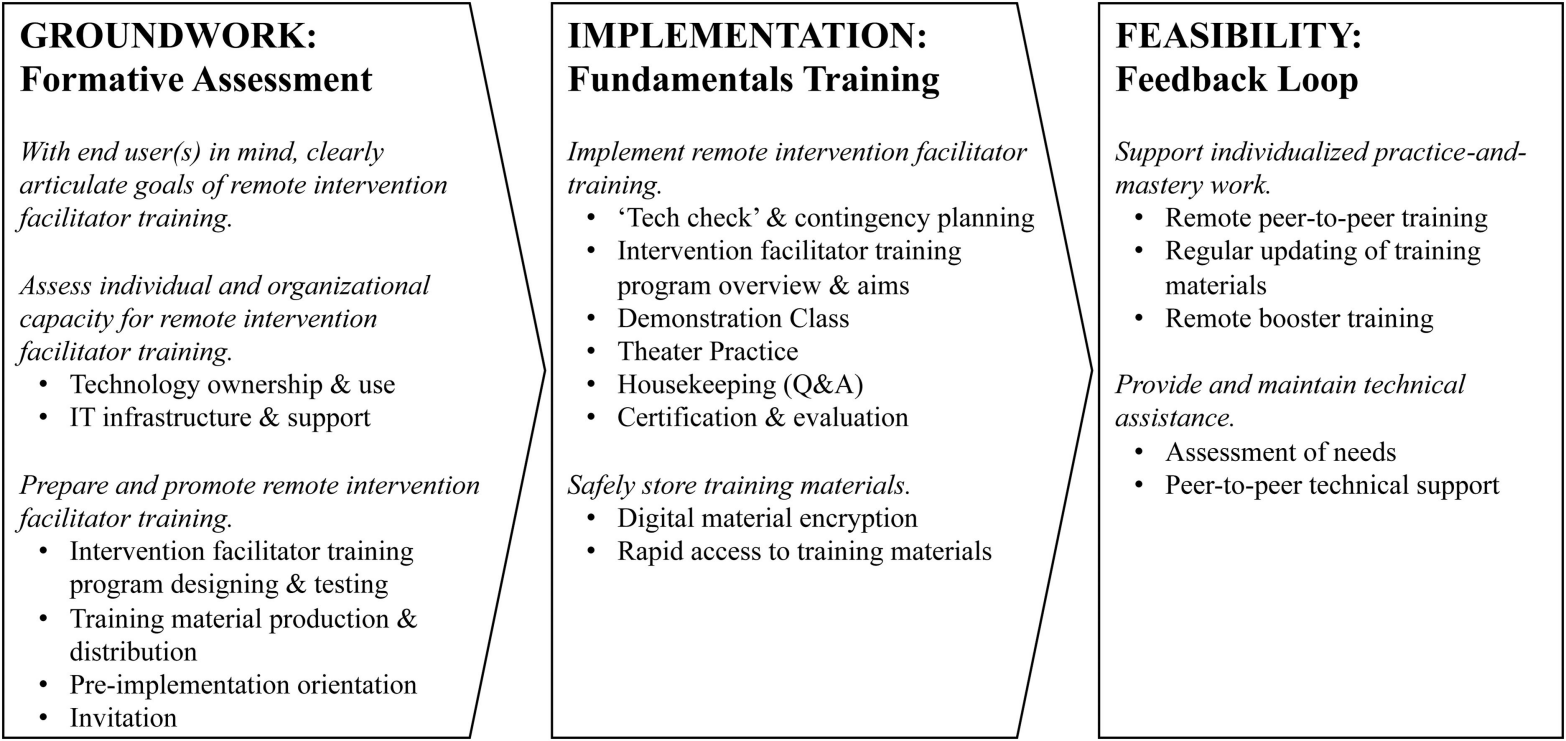
The groundwork-implementation-feasibility (GIF) model for developing and implementing remote intervention facilitator training for remote intervention delivery.

The process of developing and implementing remote intervention facilitator training—which we call the Groundwork-Implementation-Feasibility (GIF) model—involved three phases (Figure 1): ‘Groundwork’ involving formative assessment and planning of a remote intervention facilitator training program; ‘Implementation’ of the remote intervention facilitator training program focused on enhancing facilitators’ familiarity with the fundamentals of the modified intervention; and ‘Feasibility’ supporting individualized practice-to-mastery of skills involved in delivering the modified intervention through feedback and technical assistance.

## 3 Results

### 3.1 Phase 1: groundwork

***The ‘Groundwork’ phase of remote intervention facilitator training involves formative assessment and planning*.** First, *with anticipated end users in mind, we articulated goals of remote intervention facilitator training*. We determined that, for intervention facilitators, the goals of remote training are to become familiar with intervention content and delivery format, as well as to enhance confidence and proficiency in managing newly integrated digital technology, interacting remotely with intervention recipients, and addressing potential challenges that may arise during remote intervention delivery.

Next, *we assessed individual and organizational capacity for remote intervention facilitator training*. To determine the best digital platform to host a training workshop, we first surveyed facilitators about their technology ownership and use. Facilitators in this research study had access to a personal computer and some experience with internet-based computing as part of their job as well as general research and administrative tasks. Additionally, the recent shifting of team meetings and data collection activities to teleconferencing platforms (e.g., Zoom) and messenger apps (e.g., WhatsApp) had enhanced facilitators’ familiarity with telecommunications technology. These findings informed our decision to provide the training remotely and synchronously on a teleconferencing platform. Zoom was chosen for its availability of services in the languages used by the developers and the facilitators (e.g., Russian, English), features supporting interactivity among users (e.g., audio-visual functions, screen sharing, whiteboard, breakout rooms, in-meeting chat), accessibility via various common computer hardware, and low to no setup fee. Given the novelty of the modified intervention delivery and training format—thus, a potential need for additional training—we planned for the remote intervention facilitator training workshop to be held six to eight weeks prior to scheduled intervention implementation (January 2021).

Lastly, *roles and tasks of preparing and promoting the workshop were assigned*. Employing a human-centered design approach (38), the developers and the facilitators collaborated on designing a training workshop that would be easily and safely accessible from remote locations. The developers constructed and distributed a workshop agenda, along with a detailed Facilitator's Manual outlining expectations and procedures of intervention session activities, to facilitators. Activities involving collaboration among facilitators during the workshop were developed on easily accessible platforms. For instance, icebreaker games using texts or graphics used free online design tools like Canva and were built on PowerPoint slides to be viewed on Zoom via screen share; and a virtual Q&A forum was set up on Padlet where the facilitators could post questions and answers anonymously throughout the workshop. The developers worked with IT support staff from the facilitators’ workplace to host a technical assistance meeting for orienting the facilitators to hardware, software, and other devices and platforms required for the workshop. Along with reminders about the workshop, a Zoom link was sent to all facilitators ahead of time.

### 3.2 Phase 2: implementation

***The ‘Implementation’ phase of remote intervention facilitator training focuses on fundamentals training***. Following formative assessment and planning in the first phase, *we implemented the remote intervention facilitator training workshop*. Every day of the workshop started with ‘tech check’, a ritual designed to identify technical issues and develop contingency plans for overcoming unexpected technical and/or social disruptions, including limited bandwidth, service interruptions, device malfunctions, and changes in privacy levels in the attendee's environment. Technical issues were addressed through a crowdsourcing approach, whereby facilitators were invited to assist in troubleshooting them; the IT staff were also on standby to provide technical assistance. Icebreaker games were first led by the developers, and the facilitators were invited to share ideas for, and even lead, these games. During the workshop, facilitators could post questions on the Padlet-supported virtual Q&A forum (developed in Phase 1); these questions were addressed at the end of each day. We obtained verbal consent from facilitators about recording the workshop.

The workshop consisted of three modules. The first module was Demonstration Class, whereby the developers modeled delivering the intervention, from start to finish, by role-playing as a facilitator. This served as an opportunity to orient the facilitators to the updated Facilitator's Manual and introduce new or updated intervention components.

**Figure 2:**
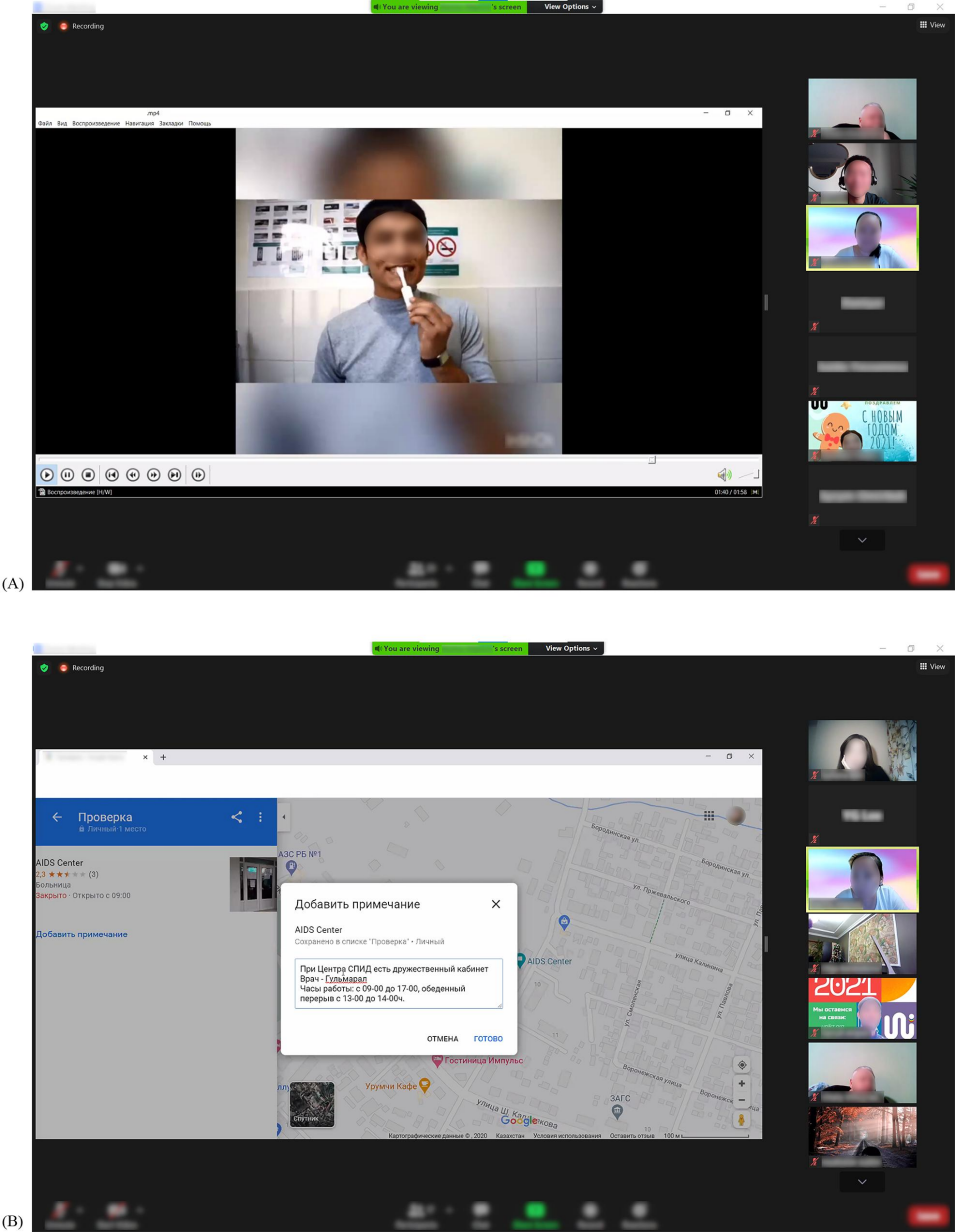
Sample activities from remote intervention facilitator training for remote delivery of *PRIDE in HIV care* via zoom: **(A)** producing and sharing a rapid HIV self-testing instruction video; **(B)** mapping SDGE-friendly HIV care services on google maps.

The second module was Theater Practice, whereby the facilitators practiced intervention delivery through role-play and real-play exercises, and received feedback from peers. The facilitators were divided into smaller groups; each group was assigned a breakout room where the facilitators took turns practicing delivering a session to a peer facilitator who role-played as an intervention recipient, as the other attendees observed. Figure 2 depicts segments of theater practice. A facilitator and a mock intervention recipient together viewed a rapid HIV self-testing instruction video (produced by study staff from Shymkent) and collaborated on customizing a map of SDGE-friendly HIV care services using Google Maps; to gather relevant information, they browsed the web and social media real-time. Also, they shared the video clip and the custom map via multiple communication channels including instant messaging services, social media platforms, and sexual networking apps.

Real-play involves facilitators experiencing and responding in real time to actual technical and interpersonal challenges (vis-à-vis role play, in which situations/roles are simulated or acted out). For our remote intervention facilitator training, real-play proved to be a powerful training tool. The following examples demonstrate the power of real-play specifically for remote training. Actual technical difficulties (e.g., sudden loss of video and/or audio, disconnection) were intentionally enacted by the developers—albeit it was not unusual for these difficulties to occur on their own accord—so the facilitators would have to implement troubleshooting and contingency measures in real-time; the real-play training value is that the facilitators would have the hands-on experience in enacting the exact same procedures and processes that they would enact if technical difficulties arose during intervention delivery, with the goal being a level of proficiency that would maintain rapport while overcoming the difficulty (39). Real-play was also used to elicit and address concerns related to technology use beyond technical difficulties, such as concerns about the security and privacy of digital platforms and services used to deliver the training and the intervention. This parallel process was used to surface both concerns as well as mitigation measures, such as avoiding the use of identifying information. Given the concerns around inherent threats or unplanned interruptions to privacy (e.g., somebody unexpectedly enters the physical room where the intervention recipient is completing the intervention), especially with stigmatized contexts and sensitive information, the remote intervention included the intervention recipient and the facilitator agreeing upon a safe word, which, when said, would result in the audio and visual transmission to convert to innocuous material or halt altogether. Real-play using safe words allowed facilitators to experience what happens if the chosen safe word was too common (e.g., the use of the safe word went unnoticed in some instances, in other instances, activity was paused unnecessarily) or too obscure (e.g., it was hard to invoke the safe word in an inconspicuous manner). This protocol reinforced wellbeing and boundaries for facilitators, but the real-play components reinforced facilitators being prepared for technical aspects of remote delivery and interpersonal complexities of delivering sensitive interventions. At the end of theater practice, the facilitators reconvened in the main room to share feedback and elucidate strategies for troubleshooting issues that might arise during remote intervention delivery. Theater practice not only offered the facilitators a chance to anticipate paralleling of various scenarios, opportunities, and challenges from the training in the actual intervention, but also generated digital files that could be used as social marketing materials during the intervention.

The third module was Administration and Housekeeping. The workshop launched with introductions of all attendees, orientation to training materials and format, and review of the study aims and ethics. The last day of the workshop involved discussion of recruitment of intervention recipients, handling common mistakes and challenging situations, time management, and clinical supervision.

The workshop concluded with a training certification ceremony, whereby the facilitators were awarded a digital badge of training completion that they could use for external communication purposes—for instance, in the email signature or as a profile photo in the apps used for participant-facing activities. Facilitators also completed an anonymous satisfaction survey that elicited numerical rating of overall satisfaction on a semantic different scale of possible scores ranging from 1 (extremely dissatisfied) to 10 (extremely satisfied) as well as textual feedback on the workshop's strengths and areas of improvement.

All 13 facilitators from Almaty, Shymkent, and Astana attended the remote training workshop for full duration, obtained the digital training completion badge, and completed the satisfaction survey. For the overall satisfaction rating, two facilitators gave a score of 10, seven a score of 9, three a score of 8, and one a score of 5; the mean score was 8.6, indicating general satisfaction with the remote training workshop.

Several strengths of the remote training workshop were reported. Most facilitators acknowledged the functional benefits of engagement afforded by the remote modality. For instance, many perceived the workshop to be accessible and convenient, while others expressed surprise and enthusiasm for being able to remotely facilitate interactive activities.

“I did not expect that it would be so interesting to participate in the online training. The warm-ups (icebreaker games) surprised me. It was very convenient that in the morning, one could participate in training from home.” (Anonymous)

In addition, some facilitators noted positive attitudes of engagement among peers, characterizing the general atmosphere of the workshop as “friendly” and “good mood.”

Several areas of improvement were also reported. One common area concerned with technical issues. Highlighting a potential challenge of having unstable connection, one facilitator shared their wish for “improved internet speed.” Another area was incorporation of physical activities. One facilitator suggested integrating activities “that don't involve sitting still.” Finally, several facilitators reported scheduling conflicts and suggested different times of a day for conducting the workshop.

These impressions served as parallel process observations, whereby facilitators could anticipate impressions of excitement and convenience among remote intervention recipients, as well as concerns about issues of internet connectivity, physical strain, and scheduling conflict.

*At the conclusion of the remote intervention facilitator training workshop, the developers safely stored training materials*. The workshop generated additional digital training materials, including video recordings, a Q&A forum, and custom maps. These materials were encrypted and stored in a secure file-sharing portal. Upon request, the materials could be rapidly retrieved and distributed by the developers.

### 3.3 Phase 3: feasibility

***The ‘Feasibility’ phase establishes feedback loop for sustaining individualized intervention facilitator training*.** *After the workshop, we supported the facilitators becoming more proficient at remote intervention delivery through on-going practice*. Facilitators engaged one another in remote, synchronous practice sessions. Supplemental materials, including recording of the workshop and the virtual Q&A forum, were made available to the facilitators, and could be reviewed asynchronously. The facilitators could also actively participate in updating training resources, such as the virtual Q&A forum. Prior to intervention implementation, two booster training sessions were provided. Both sessions were attended by all 13 facilitators who had completed the initial training workshop. Booster training sessions were shorter in duration and focused on troubleshooting common errors made by the facilitators.

*To support on-going practice, we continued providing technical assistance*. Two facilitators with topical or technical expertise were invited to aid coaching of other facilitators. The developers and the peer coaches met regularly to identify areas or individuals needing additional support. As the facilitators began delivering the intervention, they systematically provided process measures on the status and quality of intervention activities at the completion of each session; this feedback further informed future training areas.

## 4 Discussion

The purpose of our study was to describe a process of developing and implementing remote intervention facilitator training for remote delivery of a multisession HIV preventive intervention, *PRIDE in HIV Care*. The process involved formative assessment and planning that laid the groundwork for a five-day workshop; implementation of the workshop emphasizing fundamentals training in remote intervention delivery; and maintaining feasibility of remote intervention facilitator training through ongoing individualized practice and feedback. Overall, full retention of the facilitators in the workshop and relatively high satisfaction ratings illustrated feasibility of the remote training format among the facilitators of the *PRIDE in HIV Care* intervention.

### 4.1 Lessons learned

**Figure 3:**
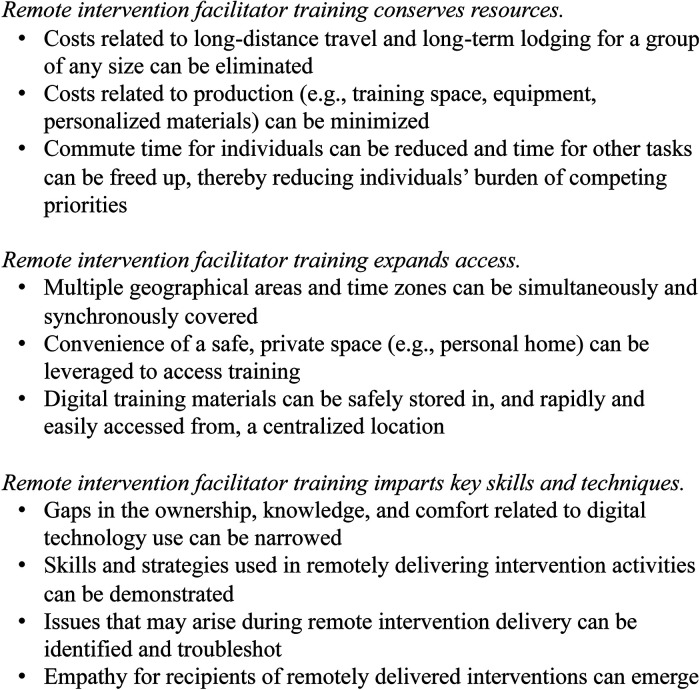
Advantages of remote intervention facilitator training for remote intervention delivery.

Several opportunities of remote training were delineated from the process of developing and implementing remote intervention facilitator training (Figure 3). First, remote intervention facilitator training helped the study conserve resources. Previously, in-person intervention facilitator training workshops incurred costs of travel and lodging of up to 20 individuals. In addition, one of the intervention developers travelled to study cities in the implementation phase regularly to provide booster training. Holding the intervention facilitator training workshop and booster training sessions remotely eliminated travel and lodging costs. Commute time was also reduced significantly; this gave the facilitators time to attend to other urgent matters and thus addressed the burden of competing priorities (40). Another set of reduced costs pertained to equipment and production. Training space and equipment that had been rented for the in-person workshops were no longer needed; instead, they were replaced by hardware and software that was already in possession and use by the facilitators. Additionally, we used free versions of services (e.g., Zoom, Padlet, Canva, Google Maps, Telegram) to host the workshop and build supplemental materials. As free digital platforms and services are particularly vulnerable to privacy and security risks (41, 42), efforts to ensure the privacy, safety, and confidentiality of users must be prioritized and maintained, especially in environments characterized by high levels of stigma; this underscores the value, perhaps the necessity, of the real-play exercises focused on concerns about the technology used for training as well as intervention delivery.

Secondly, remote intervention facilitator training expanded access to training resources. All Zoom meetings related to development and implementation of remote intervention facilitator training, including the workshop, could be accessed from virtually anywhere with internet connection. Indeed, the workshop was attended by 16 individuals from at least four cities across Kazakhstan and the United States. As supplemental materials, including recordings of all training sessions, were stored digitally, they could be accessed efficiently and asynchronously. Such flexibility with frequency and time of accessing digital training materials could be useful for providing additional training to anyone who did not attend the workshop—for instance, new hires resulting from staff turnovers.

Lastly, remote intervention facilitator training had the unique advantage of imparting key skills and techniques specific to remote intervention delivery. In developing and implementing remote intervention facilitator training, particularly the workshop, we utilized many of the same digital services incorporated in the remotely delivered intervention. We ensured that the developers and the facilitators not only had access to the same services but also gained familiarity with using them. Therefore, remote intervention facilitator training helped to narrow the gaps in ownership, knowledge, and comfort with digital technology more efficiently. Remote intervention facilitator training also enabled the facilitators to gain skills and strategies needed for remote intervention delivery. It is possible that, for the workshop, being on the same telecommunication platform as used in the remotely delivered intervention offered the facilitators a chance to observe and practice important facilitation techniques such as confirming digital accessibility and personal safety through “tech checks” and operating multiple digital services simultaneously. Facilitators could also build skills through reviewing the recordings of the workshop asynchronously or practicing with a peer facilitator synchronously. In addition to implementation skills, remote intervention facilitator training allowed the facilitators to anticipate what recipients of the remotely delivered intervention might experience through a parallel process (28). For instance, internet connectivity failures or qualms about digital technology use during the remote training workshop that some facilitators experienced served as an opportunity for the workshop attendees to acknowledge that the parallel scenarios with intervention recipients were possible.

Our experience highlights the value of mirroring, as much as possible, components of remote intervention facilitator training with those of a remotely delivered intervention. Future remote delivered training programs should intentionally design for parallel processes by exposing facilitators to the same technological, environment, and interpersonal challenges that intervention recipients will face. This includes allowing facilitators to experience and troubleshoot connectivity issues, navigate privacy concerns in user settings, and manage the emotional responsibility of discussing stigmatized topics remotely (39). Real-play exercises must extend beyond technical skills to encompass the affective dimensions of remote delivery, with reflections on how facilitators’ own experiences inform their understanding of participant needs. It is recommended to discuss how technical difficulties affect engagement and what support strategies may be helpful depending on the intervention. Remote training programs should explicitly discuss parallel processes with facilitators to help recognize how training experiences can deepen empathy and inform person-centered intervention delivery within marginalized populations (28, 43).

Despite many positive attributes, remote intervention facilitator training also presented challenges. Throughout the process of developing and implementing remote intervention facilitator training, the facilitators reported technical issues—most commonly, malfunctioning of hardware and software, including problems with internet connectivity, and varying levels of digital literacy and confidence for operating unfamiliar technologies. Technical assistance should be made available early and widely to help mitigate technical issues. Regular tech checks and contingency planning can help facilitators expediently identify and handle technical issues. Pre-workshop assessment of and orientation to technology use, as well as delegation of coaching to peer facilitators, is imperative to sustaining remote intervention delivery. Meanwhile, some facilitators suggested incorporating more physical activity in the remote training workshop. This suggestion mirrors efforts worldwide to promote physical activity in daily life during the pandemic that hand precipitated prolonged sedentary time (44). Future efforts of remote intervention training should prioritize physical and mental wellness of the trainees.

### 4.2 Limitations

Several limitations should be considered in the interpretation of findings as this piece describes a single case example of developing and implementing remote intervention facilitator training for specific HIV prevention interventions in Kazakhstan. There may also be a limit to the generalizability of the GIF model to other contexts, populations, or intervention types. The unique sociocultural contexts of SDGE populations in Kazakhstan, including specific patterns of stigma, discrimination, and technology access, may not be representative of other contexts. Definitive claims on the strongest method of intervention delivery are hard to establish, especially alongside the notion that the COVID-19 pandemic necessitated the intervention's shift to remote training. With this evolution unplanned, the ability to isolate the effects of training modality from the effects of the pandemics’ significant disruptions on vitality and life is hard to parse apart. Moreover, the sample of facilitators was small and consisted of individuals employed by the research team with prior experience in behavioral health research and community organizing, something that might not reflect the experiences of facilitators in other settings or with different backgrounds. In the end, no systematic data was collected on participant experiences of the training itself, instead relying on informal feedback and process observations that can introduce bias in reporting. Despite limitations, our research offers valuable insights for practitioners seeking to develop remote training programs for remote delivery intervention.

## 5 Conclusions

Intervention facilitator training is critically important for successful implementation of complex behavioral health interventions. We developed and implemented remote intervention facilitator training designed to improve competence and motivation in the remote delivery of an HIV prevention intervention in Kazakhstan. This study adds to a nascent literature that is largely composed of studies describing remote training for interventions delivered in-person and offers a model for developing and implementing remote training for interventions delivered remotely. Our experience demonstrates that remote training offers distinct advantages beyond cost reduction, including imparting remote specific skills, leveraging parallel processes for building empathy, and expanding access to training. The importance of intentional parallel process design, real-play exercises, ongoing technical support, and attention to facilitator wellbeing when working with stigmatized populations is a necessity. As behavioral health interventions increasingly incorporate technology like artificial intelligence or digital research platforms to adapt to hybrid or remote models, remote training is uniquely positioned to prepare facilitators for the technical, interpersonal, and ethical complexities of delivering evidence-based interventions to vulnerable populations in digital environments.

## Data Availability

The raw data supporting the conclusions of this article will be made available by the authors, without undue reservation.
